# Looking back and moving forward: can we accelerate progress on adolescent pregnancy in the Americas?

**DOI:** 10.1186/s12978-017-0345-y

**Published:** 2017-07-14

**Authors:** Sonja Caffe, Marina Plesons, Alma Virginia Camacho, Luisa Brumana, Shelly N. Abdool, Silvia Huaynoca, Katherine Mayall, Lindsay Menard-Freeman, Luis Andres de Francisco Serpa, Rodolfo Gomez Ponce de Leon, Venkatraman Chandra-Mouli

**Affiliations:** 1 Pan American Health Organization (PAHO/WHO), 525 23rd Street, NW, Washington, DC, USA; 2 University of Washington, Seattle, WA USA; 3 United Nations Population Fund (UNFPA), Regional Office for Latin America and the Caribbean, Panama City, Panama; 4 United Nations Children’s Fund (UNICEF), Regional Office for Latin America and the Caribbean, Panama City, Panama; 5 International Planned Parenthood Federation/ Western Hemisphere Region (IPPF/WHR), New York, USA; 6 Center for Reproductive Rights, New York, USA; 7 The Torchlight Collective, Nashville, TN USA; 8 Pan American Health Organization (PAHO/WHO), Washington, DC, USA; 9 Center for Perinatology, Women’s Health, and Reproduction (CLAP/PAHO), Montevideo, Uruguay; 10 World Health Organization (WHO), Geneva, Switzerland

**Keywords:** Adolescent pregnancy, Equity, Latin America and the Caribbean

## Abstract

Adolescent fertility rates in Latin America and the Caribbean (LAC) remain unacceptably high, especially compared to the region’s declining total fertility rates. The Region has experienced the slowest progress of all regions in the world, and shows major differences between countries and between subgroups in countries. In 2013, LAC was also noted as the only region with a rising trend in pregnancies in adolescents younger than 15 years. In response to the lack of progress in the LAC region, PAHO/WHO, UNFPA and UNICEF held a technical consultation with global, regional and country-level stakeholders to take stock of the situation and agree on strategic approaches and priority actions to accelerate progress. The meeting concluded that there is no single portrait of an adolescent mother in LAC and that context and determinants of adolescent pregnancy vary across and within countries. However, lack of knowledge about their sexual and reproductive health and rights, poor access to and inadequate use of contraceptives resulting from restrictive laws and policies, weak programs, social and cultural norms, limited education and income, sexual violence and abuse, and unequal gender relations were identified as key factors contributing to adolescent pregnancy in LAC. The meeting participants highlighted the following seven priority actions to accelerate progress:

1. Make adolescent pregnancy, its drivers and impact, and the most affected groups more visible with disaggregated data, qualitative reports, and stories.

2. Design interventions targeting the most vulnerable groups, ensuring the approaches are adapted to their realities and address their specific challenges.

3. Engage and empower youth to contribute to the design, implementation and monitoring of strategic interventions.

4. Abandon ineffective interventions and invest resources in applying proven ones.

5. Strengthen inter-sectoral collaboration to effectively address the drivers of adolescent pregnancy in LAC.

6. Move from boutique projects to large-scale and sustainable programs.

7. Create an enabling environment for gender equality and adolescent sexual and reproductive health and rights.

## Adolescent pregnancy as a public health and inequity issue

Pregnancy and childbearing in adolescence contribute to increased risks of maternal mortality and morbidity, especially in very young adolescents [1–3]. Babies born to adolescent mothers face increased risks of premature birth and low birth weight [1–3]. By propelling girls into motherhood before they are physically, emotionally or financially ready, adolescent pregnancy profoundly affects girls’ life trajectories, limiting their educational attainment and their earning potential, thereby increasing the likelihood of poverty and perpetuating intergenerational cycles of poverty [1–3]. Adolescent girls who are already marginalized are often disproportionately affected by early pregnancy, due to its interlinkages with poverty, social exclusion, sexual violence and child marriage, and their limited access to comprehensive sexuality education and sexual and reproductive health services including contraceptive information, counselling and services [1–5].

A recent estimate suggests that meeting the unmet need for modern contraceptives of women aged 15–19 years would globally avert 2.1 million unplanned births, 3.2 million abortions, and 5.600 maternal deaths every year [2].

Although the total fertility rate has substantially declined in LAC, adolescent specific rates have declined only slightly over the past 15 years and continue to be the second highest in the world, surpassed only by Sub-Saharan Africa [6]. In response to the relative lack of progress in the LAC region, PAHO, UNFPA and UNICEF held a technical consultation with global, regional and country-level stakeholders to take stock of the situation, reflect on hindering factors, and agree on a set of priority actions to accelerate progress in this area. This paper presents the key issues raised and discussed during the expert meeting, and summarizes the proposed actions to accelerate progress.

## What is the current situation of adolescent pregnancy in LAC?

As a region, LAC has the second highest adolescent fertility rate in the world, estimated to be 67 births per 1000 girls aged 15–19 for the period 2010–2015, compared to 51 births per 1000 girls worldwide [6, 7]. From 1980 to 2000 the adolescent fertility rate remained stagnant in LAC, followed by a slow downward trend over the past 15 years [6, 7]. In contrast, there has been a marked decline in the total fertility rate in LAC from four births per woman in 1980–1985, to 2.2 births per woman in 2010–2015 [6]. Because the total fertility rate in LAC has declined, adolescent pregnancies now constitute a larger proportion of the total number of births and the associated burden of mortality and morbidity. An estimated 15% of all pregnancies in LAC occur amongst girls younger than 20 years of age [8]. Data on pregnancy in girls younger than 15 years in LAC is limited. Based on available data from household surveys, UNFPA estimates that 2% of women of reproductive age in Latin America and the Caribbean had their first delivery before the age of 15, with LAC being the only region with an increasing trend in pregnancies in the under-15 age group [3].

When adolescent fertility in LAC is reviewed by country and sub-region, a diverse and complex picture emerges. Country-level estimates for the period 2010–2015 range from 17.2 births per 1000 girls in Guadeloupe to 100.6 in the Dominican Republic [6]. The majority of the countries with the highest estimated adolescent fertility rates in the Region are in Central America, with the highest rates in Nicaragua, Guatemala and Panama, ranging from 78.5 to 92.8 per 1000 [6]. In the Caribbean, the Dominican Republic and Guyana have the highest rates at 100.6 and 90.1 per 1000, while most of the other Caribbean countries remain below the regional average. In South America, Venezuela and Ecuador have the highest estimated adolescent fertility rates at 80.9 and 77.3 per 1000 [6].

**Fig. 1:**
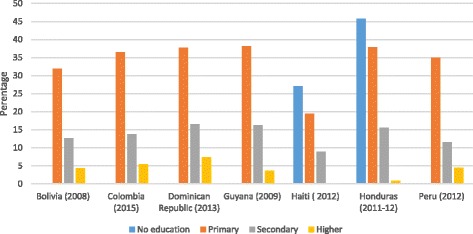
Percentage of females 15–19 years old who had begun childbearing by education level in selected LAC countries, 2008–2015

**Fig. 2:**
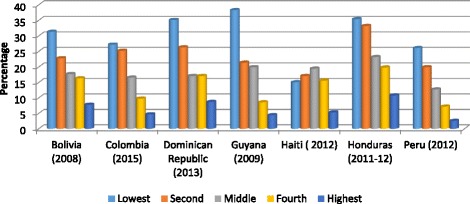
Percentage of females 15–19 years old who had begun childbearing by wealth quintile in selected LAC countries, 2008–2015

Analysis of adolescent fertility data by education level, wealth quintiles and ethnicity highlights the inequities within countries, As presented in Figs. 1 and 2, adolescent girls with no education or only primary education, and girls in the lower wealth quintiles are up to four times more likely to initiate childbearing compared with girls with secondary or higher education and girls from the highest wealth quintiles [9]. As presented in Table 1, an analysis of 2010–2011 census data from Costa Rica, Brazil, Ecuador, Mexico, Panama and Uruguay illustrates consistently higher percentages of adolescent mothers in the indigenous and rural populations in these countries [10].

**Table 1:** Percentage of adolescent mothers by age groups, place or residence, and indigenous or non-indigenous origin in the census of selected Latin American countries

Country and date of census	Age group	Percentage of adolescent mothers					
		Indigenous			Non-indigenous		
		Urban	Rural	Total	Urban	Rural	Total
Brazil, 2010	15–17 years	10.6	22.9	18.7	6.4	8.6	6.8
	18–19 years	26.8	46.9	39.4	18.2	26.6	19.5
	15–19 years	17.0	31.6	26.4	11.1	15.2	11.8
Costa Rica, 2011	15–17 years	8.5	20.3	17.0	5.3	6.7	5.7
	18–19 years	23.6	42.1	36.1	17.0	22.2	18.4
	15–19 years	15.2	28.7	24.7	10.0	12.6	10.8
Ecuador, 2010	15–17 years	9.0	9.6	9.5	8.3	11.9	9.6
	18–19 years	28.9	34.2	32.9	25.2	34.1	28.1
	15–19 years	17.4	18.5	18.3	15.0	20.3	16.8
Mexico, 2010	15–17 years	6.3	7.4	6.9	5.7	7.1	6.0
	18–19 years	23.4	27.4	25.3	20.6	25.8	21.6
	15–19 years	13.2	14.8	14.0	11.6	14.2	12.2
Panama, 2010	15–17 years	16.9	20.5	19.6	5.7	8.9	6.7
	18–19 years	38.8	54.2	49.7	19.1	28.6	21.7
	15–19 years	26.0	32.4	30.7	11.3	16.2	12.7
Uruguay, 20,100	15–17 years	6.0	4.1	6.0	4.6	4.9	4.6
	18–19 years	20.2	25.8	20.4	16.9	21.9	17.1
	15–19 years	11.6	12.5	11.6	9.3	11.3	9.4

Jorge Rodriguez Vignoli. La reproducción en la adolescencia y sus desigualdades en América Latina. Introducción al análisis demográfico, con énfasis en el uso de microdatos censales de la ronda de 2010. CEPAL, UNFPA, 2014

Meanwhile, North America has achieved major progress. Adolescent fertility rates have decreased from 51.6 births per 1000 girls in 1980–1985 to 22.3 per 1000 in the United States in 2015 [6], falling 8 percentage points in one single year from 2014 to 2015 in the USA [11]. In Canada the adolescent fertility rate dropped from 24.9 to 11.3 births per 1000 girls between 1990 and 2015 [6].

Other regions outside of the Americas also demonstrate that progress is possible at much faster rates. In South Asia, for example, the adolescent fertility rate has declined from 88 births per 1000 girls in 1990 to 47 births per 1000 girls in 2015 and the total fertility rate has declined from 4.3 births per woman in 1990 to 2.6 births per woman in 2014 [6, 7].

## What are the reasons for the slow decline of adolescent pregnancy in LAC?

Adolescent pregnancy does not occur in a vacuum; a confluence of multi-layered factors at the individual, interpersonal, community, and societal levels contribute to the occurrence and distribution of early pregnancy [2–5]. At the individual level, pregnancy in adolescent girls is often not a result of deliberate choice, but rather resulting from limited or lack of information on sexual and reproductive health and restricted or limited access to sexual and reproductive health services, including effective contraception [2–4]. The lack of access to emergency contraception even in the context of rape or incest is of particular concern [2–4]. At the interpersonal level, sexual violence and gender norms about power and control undermine girls’ agency and their ability to prevent unwanted pregnancy [2–4]. At the community level, the reluctance of gate-keepers such as parents, school teachers, as well as political, community and religious leaders to acknowledge that adolescents are sexually active impedes efforts to equip them with information, skills, and tools (such as condoms) to avoid sexual and reproductive health problems [2–4]. Additionally, the status of motherhood as a cultural value or as a pathway out of poverty can lead to early marriage or informal unions and to greater acceptance of early pregnancies [2–5].

The lack of systematic and ongoing analysis of the implementation and impact of interventions in general and related to specific groups makes it challenging to define more precisely which factors hindered progress in LAC. Factors identified by the meeting participants that may have hindered or delayed actions for prevention of adolescent pregnancy in LAC include a lack of acknowledgement of the vulnerability of girls to abuse and gender-based violence, under-investment in the human capital of girls and women, and inequalities based on gender, wealth, and educational and employment attainment that affect the aspirations and opportunities of - and for - young girls; and national-level policies that restrict the provision of comprehensive sexuality education and sexual and reproductive health services for adolescents, including contraceptive services; and finally financial and personal constraints (including biases) that hinder the application of policies – even when they are supportive [12].

Anecdotal information and occasional studies suggest that there is no single portrait of a teenage mother in LAC, but rather a variety of scenarios that operate at the same time and contribute to the current situation [5, 9, 13]. For some girls, pregnancy is unintended or unwanted, while others choose to become pregnant as an opportunity to gain adult status and upward social mobility. Some pregnancies are prompted by traditional expectations for young women to prove their fertility and cultural understandings of motherhood as an esteemed condition. For others still, pregnancy results from sexual violence or sexual abuse. Factors identified in these studies as contributing to lack of progress include limited or lack of knowledge about their sexual and reproductive health and rights, poor access to effective methods of contraception, including long acting reversible contraceptives (LARCs) and emergency contraception, lack of or improper use of contraceptive methods, the absence of, or barriers to access comprehensive post-rape care and safe abortion services, restrictive laws and policies or restrictive practices in the presence of enabling laws and policies, on providing comprehensive sexuality education, contraceptive information and services, and safe abortion care [5, 12–15].

## What has been done to address adolescent pregnancy in LAC?

While there have been regional, sub-regional and national responses to address adolescent pregnancy in LAC, they have not yet succeeded in achieving substantive progress. For example, all the sub-regions have developed multi-country plans or strategies, including the Andean Plan to Prevent Teen Pregnancy (ORAS/CONHU. Plan Andina para la prevencion del embarazo en adolescentes. 2007–2013. Unpublished document), the Strategic Plan for the Prevention of Adolescent Pregnancy in Central America and the Dominican Republic [16], the Integrated Strategic Framework for the Reduction of Adolescent Pregnancy in the Caribbean (CARICOM and UNFPA. Integrated Strategic Framework on the Reduction of Adolescent Pregnancy. Unpublished document), and an unpublished multi-country strategic framework for prevention of adolescent pregnancy developed jointly by the Southern Cone countries. These plans and strategies provide policy and operational platforms for sub-regional collaboration and generating political momentum. Several countries, including the Dominican Republic, Peru, Mexico, and Honduras are also implementing national adolescent pregnancy prevention strategies and plans [17–20].

In addition, the LAC Ministers of Health and Education adopted the Mexico Ministerial Declaration: “Educating to Prevent” in 2008, committing to improve access to and the quality of comprehensive sexuality education for young people, with the goal to reduce sexual and reproductive health risks [21]. The first session of the Regional Conference on Population and Development in Latin America and the Caribbean held in 2013 generated the Montevideo Consensus on Population and Development, which called for investing in young people through specific public policies, and articulated the regional commitment to effectively implement comprehensive sexuality education from early childhood, provide quality sexual and reproductive health services for adolescents and young persons that respond to their needs, introduce or strengthen policies and programmes to prevent pregnant adolescents and young mothers from dropping out of schools, and eliminate unsafe abortions [22].To date, the impact of these regional, sub-regional and country efforts has been limited, and adolescent fertility rates have remained stagnant in most countries and reduced slightly in others [6, 23]. For instance in Colombia the percentage adolescents aged 15–19 years who are mothers or pregnant dropped from 20.5% in 2005 to 19.5% in 2010, and to 17.4% in 2015 [23]. However, the shortage of systematic documentation and research on the issue in the region makes it difficult to understand why these efforts have not generated better results. A recent systematic review on interventions to prevent unintended and repeat pregnancy among young people in low- and middle-income countries found very few reports from the LAC region [24]. Nevertheless, this is beginning to change with some study reports and case studies published in peer-reviewed journals and in-depth analysis conducted by regional partners [25–28].

## What can we do to accelerate progress?

Through extensive discussion over the course of the two-day meeting, the group identified seven priority action areas for accelerating progress on adolescent pregnancy in LAC, described below.

1. Make adolescent pregnancy, its drivers and impact, and the most affected groups more visible with disaggregated data and stories.
  Enhanced data collection and evidence-gathering, both quantitative and qualitative, is critical to understand which population groups are most affected by adolescent pregnancy, what the drivers are, and what can be done to effectively address these drivers. Data must be disaggregated by age, ethnic group, residence, education, employment and other socio-economic characteristics, in order to help inform and shape policies and programmes at the national and sub-national levels to tailor them to local realities. Furthermore, the effects of unwanted pregnancy on individual girls’ lives, especially in girls younger than 15 years old, must be made more visible through, for examples, real life stories in the media. These efforts can help to reach decision makers and the public at large in order to gain support for investment and action.

2. Design interventions targeting the most vulnerable groups, ensuring the approaches are adapted to their local realities and address their specific challenges.
  The LAC region is one of the most culturally diverse and inequitable regions of the world, and the factors fuelling adolescent pregnancy take on different weights and forms between, and within countries. In line with the Sustainable Development Goals (SDGs) [29] and the Global Strategy for Women’s, Children’s and Adolescent’s Health [30], regional and country-level efforts must ensure that investments reach the most vulnerable adolescents first through the application of equity-based and culturally appropriate approaches.

3. Engage and empower youth to contribute to the design, implementation and monitoring of strategic interventions
  Meaningful adolescent and youth engagement in the design, implementation and monitoring of laws, policies and programmes aimed at realizing their sexual and reproductive health and rights is critical for ensuring that interventions address the local realities of adolescents. When adolescents are at the decision-making table as equals with other critical stakeholders, and are respected and supported to share ownership, resulting interventions are more likely to respond to their needs. The rights of adolescents both to make well-informed and well-supported decisions about their own health and wellbeing, and to contribute meaningfully to policy and programmatic decisions that affect their lives, must underpin all efforts.

4. Abandon ineffective interventions and invest in applying proven ones
  There is an urgent need for the adoption and implementation of a multi-sectoral package of interventions that have shown promise of effectively addressing adolescent pregnancy, including comprehensive sexuality education, informing and empowering adolescents to make well-informed decisions about their sexual and reproductive health, increasing access to LARCs and other modern contraceptives, building community understanding and support about pregnancy prevention in adolescents, preventing marriage before 18 years, preventing sexual violence and coercion, and economic and social empowerment programmes for adolescent girls [30, 31]. The package of interventions should be tailored to the local political, social, and cultural context. Table 2 provides examples of evidence-based interventions applying an ecological perspective.
  However, many countries and programmes continue to use interventions that have been proven to be ineffective. For instance, despite the fact that programmes like ‘abstinence-only education’ have been proven ineffective in equipping young people with the knowledge and skills to prevent unwanted pregnancy, they continue to be included in some country strategies and to receive political support and corresponding financing.
  The expert group put out a specific call to abandon interventions where there is evidence demonstrating inefficacy and a recommendation to invest in the implementation of proven interventions with intensity and over a sustained period [32]. Further, it called for multi-pronged interventions that focus simultaneously on multiple specific outcomes (e.g. providing adolescents with contraceptive information and services, working to change community norms and attitudes on adolescent sexuality, and putting in places laws and policies that legitimize such programming in line with international human rights treaties, recognizing adolescents’ rights) and with strong systems for monitoring and evaluation [32].
  Similarly, more efforts are needed to generate evidence on what works in the region to prevent early pregnancy, with a particular focus on those who are most marginalized (e.g. Afro-descendants, indigenous, poor, out-of-school adolescents), in order to produce a critical mass of evidence.

5. Strengthen intersectoral collaboration to effectively address the drivers of adolescent pregnancy in LAC
  Intersectoral collaboration is widely recognized as critical for successfully preventing adolescent pregnancy, but is rarely implemented to the necessary extent. Instead of working in sector-based silos, well-planned and continuously monitored mechanisms to foster complementary activities should be established. Within each sector, all players who could make meaningful contributions – government bodies, nongovernment organizations and civil society bodies (including those working for and with young people and run by young people themselves) – should be involved, using robust mechanisms. One tangible example is that both health and education sectors must roll out comprehensive sexuality education for in and out-of-school adolescents along with efforts to reach them with the sexual and reproductive health services they need [25, 32].

6. Move from boutique projects to large-scale and sustainable programmes
  Programmes that are generating desired results are often caught in the pilot phase, and efforts may be piecemeal and scattered, often not aligned with institutional systems and structures that can foster scale-up and institutionalization. Similarly, programmes that have been scaled up successfully, such as comprehensive sexuality education and adolescent-friendly health services programs in Argentina and Colombia, often need intensified efforts to be strengthened and sustained in order to have lasting impact [24, 25].

7. Create an enabling environment for gender equality and adolescent sexual and reproductive health and rights.
  As mentioned, adolescent pregnancy is caused by a complex web of factors operating at different levels of the ecological framework. To accelerate progress in the reduction of adolescent pregnancy, interventions must address all these levels simultaneously [31]. This can be accomplished by developing and implementing packages of interventions that adapt globally recognized standards and best practices to national and local contexts, and include clear results frameworks and time frames, as well as monitoring and evaluation measures. These packages of actions must utilize a rights-based approach to adolescent health and prioritize gender equality and the elimination of gender stereotypes.
  Additionally, greater attention should be devoted to promoting civil society engagement to build a movement of support for adolescents’ rights, generally, and their sexual and reproductive health and rights, and ending sexual violence, specifically. Members of the community, including parents, teachers, and religious leaders, all play important roles in keeping adolescents safe and creating opportunities for their futures. Yet, there is a considerable lack of awareness-raising and sensitization around adolescent sexuality among these groups. Further, a robust civil society movement is essential for overcoming political stagnation on this issue, and utilizing accountability mechanisms to remediate injustices.

**Table 2:** Examples of interventions to improve contraceptive access and uptake by adolescents across the ecological framework^a^

Level of ecological framework	Examples of intervention
Individual	- Educate adolescents about contraception
Relational	- Encourage and support communication on contraception between couples, within and outside unions.
Community	- Build community support for contraceptive provision to adolescents - Make health service provision more responsive and friendly to adolescents
Societal	- Legislate access to contraceptive information and services - Reduce the cost of contraceptives to adolescents

^a^These evidence-based interventions are taken from WHO’s Guidelines for preventing early pregnancy and poor reproductive outcomes to adolescents in developing countries

## Conclusion

There was widespread consensus among the meeting participants, representing a variety of stakeholder groups in the LAC community, including Ministries of Health and Education, multilateral and bilateral partners, civil society and young people, on the need to act now to address adolescent pregnancy in the Region in a more concerted manner, employing multi-faceted evidence-based approaches tailored to local needs, and focusing on quality and equity. The meeting called for implementation of the political commitment articulated in global and regional instruments, to provide the support necessary to galvanize the intersectoral action needed to accelerate progress. This call to action is well-aligned with the Sustainable Development Goals (SDGs) and the Global Strategy for Women’s, Children’s, and Adolescent Health which stress that the prevention of adolescent pregnancy will contribute not only to improvements in the survival of adolescents, but also essential for them to thrive, to prevent the intergenerational transmission of marginalization, and maximize the contribution of girls and women to the wellbeing and development of their communities.

*Some of the authors are staff members of the Pan American Health Organization. The authors alone are responsible for the views expressed in this publication, and they do not necessarily represent the decisions or policies of the Pan American Health Organization.*

## Abbreviations

LAC: Latin America and the Caribbean
LARCs: Long-Acting Reversible Contraceptives
PAHO: Pan American Health Organization
PLANEA: Andean Plan to Prevent Teen Pregnancy
SDGs: Sustainable Development Goals
UNFPA: United Nations Population Fund
UNICEF: United Nations Children’s Fund
WHO: World Health Organization
